# Retraction Note: Systematic evaluation of percutaneous radiofrequency ablation versus percutaneous ethanol injection for the treatment of small hepatocellular carcinoma: a meta-analysis

**DOI:** 10.1186/s40001-015-0131-7

**Published:** 2015-03-26

**Authors:** Rong-Hua Xu, Wei Gao, Chao Wang, De-Kai Guo, Lin Tang, Hui Zhang, Cong-Jun Wang

**Affiliations:** Department of Oncology Surgery, The Affiliated Hospital of Hainan Medical College, 31 Longhua Road, Haikou, 570102 Hainan Province China; Department of Hepatobiliary and Pancreatic Diseases, Shanghai East Hospital, Tongji University School of Medicine, 150 Jimo Road, Pudong New District Shanghai, 200120 China; Department of Surgery, University of Michigan Medical School, 1500 E Medical Center Dr., Ann Arbor, 48105 MI USA; Translational Oncology Program, University of Michigan Medical School, 1600 Huron Parkway, Ann Arbor, MI 48109 MI USA

## Retraction

The Publisher and Editor regretfully retract this article [1] because the peer-review process was inappropriately influenced and compromised. As a result, the scientific integrity of the article cannot be guaranteed. A systematic and detailed investigation suggests that a third party was involved in supplying fabricated details of potential peer reviewers for a large number of manuscripts submitted to different journals. In accordance with recommendations from COPE we have retracted all affected published articles, including this one. It was not possible to determine beyond doubt that the authors of this particular article were aware of any third party attempts to manipulate peer review of their manuscript.
